# Personalia

**Published:** 1914-05-16

**Authors:** 


### Personalia.

Lord Wolverton has accepted the presidency of the Royal Hospital for Incurables, Putney Heath.

Sir William Crawford has been elected to represent the Royal Victoria Hospital, Belfast, on the senate of Queen's University.

Mr. H. F. Keene has been appointed clerk of the Asylums and Mental Deficiency Committee of the London County Council at a salary of £1,025 a year.

Mr. Ernest Collins, who died recently at Bournemouth, had been for three years a vice-president of the Hampstead General and North-West London Hospital. On the motion of Councillor E. A. O'Brien, Mayor of Hampstead, the Council has passed a vote of condolence with Mrs. Collins.

Mr. Cyril Cobb, chairman of the Asylums and Mental Deficiency Committee of the London County Council, and Mr. F. R. Anderton, a former chairman of the Public Health Committee, with Mr. H. F. Keene, are to represent the County Council at the Sixth International Congress on Social Works Service to be held in London next year.

